# Internet-based language production research with overt articulation: Proof of concept, challenges, and practical advice

**DOI:** 10.3758/s13428-021-01686-3

**Published:** 2021-11-19

**Authors:** Anne Vogt, Roger Hauber, Anna K. Kuhlen, Rasha Abdel Rahman

**Affiliations:** 1 Humboldt-Universität zu Berlin, Rudower Chaussee 18, 12489 Berlin, Germany; 2 Berlin School of Mind and Brain, Berlin, Germany

**Keywords:** Language production, Voice onset latency, Online experiments, Overt articulation, Picture, word interference

## Abstract

Language production experiments with overt articulation have thus far only scarcely been conducted online, mostly due to technical difficulties related to measuring voice onset latencies. Especially the poor audiovisual synchrony in web experiments (Bridges et al. 2020) is a challenge to time-locking stimuli and participants’ spoken responses. We tested the viability of conducting language production experiments with overt articulation in online settings using the picture–word interference paradigm – a classic task in language production research. In three pre-registered experiments (*N* = 48 each), participants named object pictures while ignoring visually superimposed distractor words. We implemented a custom voice recording option in two different web experiment builders and recorded naming responses in audio files. From these stimulus-locked audio files, we extracted voice onset latencies offline. In a control task, participants classified the last letter of a picture name as a vowel or consonant via button-press, a task that shows comparable semantic interference effects. We expected slower responses when picture and distractor word were semantically related compared to unrelated, independently of task. This semantic interference effect is robust, but relatively small. It should therefore crucially depend on precise timing. We replicated this effect in an online setting, both for button-press and overt naming responses, providing a proof of concept that naming latency – a key dependent variable in language production research – can be reliably measured in online experiments. We discuss challenges for online language production research and suggestions of how to overcome them. The scripts for the online implementation are made available.

## Introduction

### Reasons for conducting online language production experiments

Many psychological experiments based on behavioral measures can be run online. This brings great advantages in comparison to lab-based testing. For example, online experiments facilitate testing larger samples, promoting science to a larger community, and potentially consuming less resources during data collection (e.g., Grootswagers, 2020). This greater efficiency in data collection has led to the replication and extension of many behavioral paradigms in online settings. Even experiments which require precise measures of reaction times can reliably be conducted on the web (e.g., Anwyl-Irvine, Dalmaijer, Hodges, & Evershed, 2020a; Anwyl-Irvine, Massonnié, Flitton, Kirkham, & Evershed, 2020b; de Leeuw, 2015; Gallant & Libben, 2019; Hilbig, 2016; Pinet et al., 2017). Furthermore, there is an increasing awareness for the need to test more diverse populations in order to raise the external validity of experimental findings (Speed et al., 2018). Web-based testing is one of the options to ensure that our understanding of the human mind extends to the population at large. Most recently, the popularity of web-based testing has been gaining additional momentum as the COVID-19 pandemic forces researchers to think of alternatives to lab-based testing (Sauter, Draschkow, & Mack, 2020).

Within psycholinguistics, language production research is so far underrepresented when it comes to online-based testing. To the best of our knowledge, typical language production tasks such as picture naming, during which participants’ overt articulatory responses are acquired in order to determine voice onset latencies, have so far not been investigated in online settings. In this study, we provide a proof of principle for deriving voice onset latencies from recordings of overt articulatory responses time-locked to pictorial stimuli. To this end, we implemented the picture–word interference (PWI) task, a classic paradigm to investigate lexical access during language production (for a recent review see Bürki et al., 2020), in an online version. We demonstrate the viability of this approach by comparing voice onsets computed from short audio recordings of overt naming responses with a manual classification task providing response times depending on manual keyboard button-press responses. In a direct comparison, we show similar interference effects typically observed in lab-based picture–word interference studies for both overt picture naming responses and for manual button-press classifications of the picture names. Furthermore, we provide practical advice on moving language production research online.

### Current challenges when running language production studies online

There are several challenges to conducting online language production research relying on overt naming responses. First, in the lab, the technical equipment (e.g., microphones, sound shielded booths) ensures a high quality of the acquired speech data and technical requirements are kept constant within and across participants. In an online study, participants need a microphone and need to explicitly grant access in order to record speech as a dependent variable. This approval process can disrupt the experimental procedures at different time points – depending on the individual browser’s security settings. Furthermore, recording quality may differ widely between participants due to technical reasons or background noise. Second, in the lab, the experimenter would typically monitor if the participant is complying with the instructions, e.g., naming the pictures presented on the screen in a correct manner. However, there is no easy way to monitor the performance of participants’ online verbal responses. In an online language production experiment, recorded verbal responses can only be checked after completion of the experiment. Third, lab-based experiments have the option of using voice keys, special hardware devices for automatically detecting the onset of vocal responses. Alternatively, or in addition, in the lab vocal responses can be recorded as audio files, which are scanned for the start of speech in a subsequent step after the experiment. Voice onset latencies are a key dependent variable in many language production experiments and result as the latency between the onset of a picture presentation and the onset of the naming response. However, none of the available tools for running online experiments offers the possibility to determine voice onset latencies instantly and directly log them, rendering online language production research potentially more laborious after data acquisition.

Lastly, and perhaps the most serious challenge, is to precisely timelock vocal responses to certain events, e.g., the visual presentation of a picture on a screen, with little or no variation within and between participants. In the lab, experimenters use specific hardware and software to control for audiovisual synchrony, ensuring that the timing between stimulus presentation and voice recording is reliable. Unfortunately, to date, none of the packages or programs for running online experiments offers an option for recording overt articulatory responses precisely time-locked to a (visual) stimulus. Only very recently has there been some development in this area resulting in beta versions of experimental software with audio recording possibilities and there seems to be a lively development process surrounding these versions (e.g., see the Gorilla Audio Recording Zone, or the jspsych-image-audio-response-plugin by Gilbert, 2020). However, none of these versions can, to date, ensure the high audiovisual synchrony which would be needed in order to test the typically investigated effects in language production research, which sometimes rely on mean voice onset differences in the range of a few milliseconds. To date, a published validation of their timing properties is still missing.

A recent meta-analysis compares a range of experiment builders concerning the reliability of synchronous presentation of visual and auditory stimuli, both lab-based and online, and testing different operation systems and browsers (Bridges, Pitiot, MacAskill, & Peirce, 2020; Reimers & Stewart, 2016). These studies demonstrate that the lag between visual and auditory onset varies considerably. As audio output timing, i.e., the start of audio recordings, relies on specific hardware and software properties as well, it can be assumed that it is likewise difficult to control for a precisely stimulus-locked onset of an audio recording in online settings where a large number of participants with different computer and browser configurations take part. Bridges et al. (2020) conclude that for online experiments, none of the tested packages can guarantee reliable audiovisual synchrony. They argue that JavaScript technology, which is the basis for most web-based experiments, would need to be improved in order to obtain precisely timed audio measures in the millisecond range. However, precisely timelocking overt articulation to pictorial stimuli might be an essential prerequisite to ensure replicability of language production paradigms in an online setting (Plant, 2016).

For the current study, as a proof of concept, we adopted a pragmatic approach to these challenges. As Bridges et al. (2020) point out, solving the problem of poor audio-visual synchrony in online software technology is not a task for the scientific user but rather for the community of software developers working with JavaScript. In the meantime, however, we aim for a “good-enough” approach, i.e., answering the question if the current methods are reliable *enough* to detect mean differences in classic paradigms and to replicate classic effects. Even for measurements made with instruments with poor resolution, mean differences can be successfully detected when aggregating over a large enough sample size (Ulrich & Giray, 1989). Therefore, given a sufficiently large number of trials and participants, one might be able to detect differences in naming latencies recorded in online settings in spite of the multiple challenges and limitations of web technology (Brand & Bradley, 2012). The same approach applies, to some extent, more broadly to all experimental research conducted online: while the data collected in these settings will invariably show some increased noise due to lack of experimental control and limitations introduced by variable personal hardware (e.g., laptop keyboards), effects should still be, and indeed are, detectable with sufficient power (Mathot & March, 2021; Pinet et al., 2017).

### Testing an online implementation of the picture–word interference paradigm with verbal and manual responses

Given the many advantages of online experiments, the aim of the present study is to test whether a robust and well-replicated, but relatively small effect in language production can be replicated in an online implementation of the task including overt naming responses. In the picture–word interference paradigm, participants name pictures while ignoring simultaneously presented distractor words. Naming latencies in this paradigm depend on the semantic relation between the distractor and the target picture, with increased naming times for semantically related versus unrelated distractor words (e.g., Lupker, 1979; Schriefers et al., 1990). This well-replicated semantic interference effect has been interpreted as a marker for the cognitive processes underlying lexical access (Bürki et al., 2020). Analyzing results from 162 studies with a Bayesian meta-analysis, Bürki et al. (2020) demonstrated that the semantic interference effect amounts to 21 ms with a 95% credible interval ranging from 18 to 24 ms. Therefore, replicating this small but robust effect in an online setting would demonstrate the viability of running time sensitive language production experiments online.

Crucially, semantic interference has not only been demonstrated for overt naming responses. A comparable effect has also been observed for a manual button-press classification task in which participants are asked to identify the last letter of the picture name as a vowel or a consonant by pressing one of two response buttons (Abdel Rahman & Aristei, 2010; Hutson, Damian, & Spalek, 2013; Tufft & Richardson, 2020). The similarity of semantic interference in vocal and manual naming responses allows us to directly compare these effects in an online implementation of the paradigm. While manual responses can more readily be implemented and recorded in online settings, we can compare the effects in this response modality directly to the recording of overt naming responses.

To this end, we adopted the design of the study by Abdel Rahman and Aristei (2010) in which participants received both versions of the task with the experimental manipulation of semantic relatedness as a within-subject factor. In this way, manual button response times can serve as a benchmark for a potential effect in the vocal onsets in audio responses. For naming latencies, we recorded audio files for each naming trial, time locked to the onset of picture presentation. Naming latencies were then computed offline. To enable online voice recording, we customized freely available tools for audio recordings on the web and included them in two different experiment builder programs. It was our primary goal to find a working solution for running language production online. Therefore, we conducted the same experiment in two different implementations to increase chances for finding a working solution. The first version was programmed and hosted in SoSciSurvey (Leiner, 2019), a platform used for conducting social and behavioral research in Germany in combination with an audio recording function based on RecordRTC (Khan, 2020). The second implementation was programmed using jsPsych (de Leeuw, 2015) with a custom audio recording plugin relying on Recorderjs (Diamond, 2016) and hosted on JATOS (Lange et al., 2015).The methods and predictions of this work have been preregistered on AsPredicted.com (AsPredicted#: 43871, available for viewing under https://aspredicted.org/blind.php?x=6ma52w). Data and analysis scripts are available at OSF (https://osf.io/uh5vr/?view_only=229679aa33604aa2a5cb400eab62099). The comparison between the two implementations is an exploratory analysis which was not preregistered.

## Experiment 1

### Methods

One version of the experiment was implemented in SoSciSurvey (Leiner, 2019) and the other version was implemented in jsPsych (de Leeuw, 2015). The two versions of the experiment (labeled *SoSciSurvey* and *jsPsych1*, respectively) were nearly identical regarding design and procedure. If not specified otherwise the information applies to both versions.

#### Participants

In the *SoSciSurvey* version, a total of 116 native German speakers between 18 and 35 years were recruited over the commercial platform Prolific (www.prolific.co.uk) and completed the experiment. They were included in the final sample when meeting all inclusion criteria until the final sample consisted of 48 participants as determined in the preregistration (21 females, 18–33 years, M_age_ = 25.71, SD_age_ = 4.28) (see also the section on Data Exclusion in Data Analysis for details on the criteria for inclusion in the final sample). The sample size was determined via an a priori power analysis using the *simr* package (Green & MacLeod, 2016). *Simr* uses simulation to estimate power, by simulating data for which the user can define the parameter estimates. We estimated the power for the overt naming task but needed to rely on estimates from a study employing the PWI paradigm (Lorenz et al., 2018) that used LMMs to analyze their data, which Abdel Rahman and Aristei (2010) did not. The resulting suggested sample size for a power estimate of 80% was 36, but we anticipated a need for more power in online studies and had decided a priori to increase the estimated sample size by one-third, thus amounting to the sample size of *n* = 48.

In the *jsPsych1* version, a total of 108 native German speakers between 18 and 35 years were recruited over the commercial platform Prolific (www.prolific.co.uk) and completed the experiment. They were included in the final sample when meeting all inclusion criteria until the final sample consisted of 48 participants as determined in the preregistration (24 females, 18–33 years, M_*age*_ = 26.06, SD_age_ = 3.99).^1^

Participants provided informed consent to their participation in the study. The study was conducted on the basis of the principles expressed in the Declaration of Helsinki and was approved by the local Ethics Committee. Participants received monetary compensation distributed via the platform Prolific.

#### Materials

The stimulus set consisted of 40 black-and-white line drawings of common objects, all of which have frequently been used in lab-based picture-naming studies in our group. Half of the German words for these objects ended in a vowel, the other half in a consonant. For the related condition, each drawing was assigned a semantically related distractor word that was not part of the response set. For the unrelated condition, the same distractor words were reassigned to different drawings to which they were not semantically related. In both conditions, half of the assigned distractors matched the name of the drawing with regards to the type of the last letter (vowel vs. consonant) and the other half did not. Thus, response compatibility of the distractor and the target regarding the classification of the last letter was balanced across the stimulus set. In the case of a compatible match, the last letters were never identical, but only matched concerning the type of letter, i.e., vowel or consonant. The line drawings were presented together with the visual distractor words that were superimposed without obscuring the visibility of the object. See online Supplementary Material A for a table of the stimulus set (https://osf.io/uh5vr/?view_only=229679aa33604aa2a5cb400eab62099).

#### Design

The experiment consisted of a 2 x 2 design with the within-subject factors *task* (button-press vs. overt naming) and *relatedness* (related vs. unrelated distractors). The dependent measure was response latency (of the button-press and the overt naming, respectively). The order of the tasks (button-press – overt naming vs. overt naming – button-press) and the assignment of buttons to responses (*p* for vowel, *q* for consonant vs. *p* for consonant, *q* for vowel) was counterbalanced across participants.

#### Procedure

At the start of the experiment, participants were given general instructions and then a preview of all 40 drawings with the corresponding names (but *without* distractors) to familiarize participants with the stimulus set. Instructions for the first task were then presented including a catch trial to ensure participants read the instructions carefully, followed by four practice trials. Each main task consisted of 80 trials showing all 80 stimuli in random order. Each trial started with a 500-ms fixation cross at the center of the screen. The stimulus, a line drawing with a superimposed distractor word, then appeared at the center of the screen (200 x 200 pixels) for a total of 2000 ms, followed by a blank screen for another 1000 ms before the next fixation cross appeared. In the button-press task response, labels (e.g., “Q consonant”, “P vowel”) were shown below the stimulus to the left and right, respectively. Once a button was pressed, the corresponding response label was highlighted but the stimulus remained on screen for the full duration of the trial. In the overt naming task, audio files were recorded for each trial starting at stimulus onset, producing 80 recordings with a predefined duration of 3000 ms for each participant. There was a short break after the first task before the instructions of the second part were presented. When both tasks were completed, participants received debriefing information and were then linked back to the website of Prolific in order to validate their participation.

#### Technical Implementation of audio recording

The technical implementations for both experimental platforms relied on JavaScript. JavaScript is a programming language that forms, together with HTML and CSS, the core technology of the Internet. Importantly, all modern browsers rely on JavaScript and therefore no prior installation of the language itself is necessary neither on the programmers’ nor the users’ side. The implementations in our study build on APIs (application programming interfaces), which can be thought of as ready-made tool sets allowing for certain functionalities to be used. The access to participants’ microphones and the streaming of their voice input is realized via such APIs in both implementations.

The experimental platform SoSciSurvey (Leiner, 2019) is based mainly on the programming language PHP, but JavaScript code can be implemented in the functionalities provided by SoSciSurvey, as we did in our study. For the implementation of the audio recording, we included a JavaScript-based function within each audio trial. This function captures the participants’ audio input, presents the visual stimulus and starts an audio recording at the same time. The audio input is then saved in the browser’s native file format (e.g., .ogg or .webm) and transferred to the SoSciSurvey server. The JavaScript plugin *RecordRTC.js*, which we used for this purpose, is provided by Khan (2020) who provides and actively maintains a wide range of readymade JavaScript applications under the open WebRTC (web real-time communication) standard.

In our jsPsych version, the functionality of the experiment timeline relies on the experiment library jsPsych (de Leeuw, 2015), while the data are saved via a server specified by the experimenter, in our case a server based at our institute set up with JATOS (Lange, Kühn, & Filevich, 2015). For the technical implementation of audio recording within jsPsych, access to participants’ microphones is granted only once at the beginning of the overt naming part and remains permanently active during the overt naming task. The recordings are started within each trial upon stimulus presentation. Then files are immediately saved in wav format and transferred to the server. Unlike the SoSciSurvey implementation, the recording in jsPsych relies on the JavaScript plugin *recorder.js* provided by Diamond (2016), which we used to customize a jsPsych plugin to enable audio recording. Note that even though the functionality of *recorder.js* builds the base of *RecordRTC.js* (as implemented in the SoSciSurvey implementation described above), and also of other audio recording plugins, it is not actively maintained and therefore might not be working in the future, e.g., if browser standards change.

The main difference with regard to the implementation of the audio recording is that our custom jsPsych plugin uses a Web Worker API during the recording and saving of audio files. Web Workers allow to run tasks in the background without interfering with the user’s interface. This should ensure, for example, that the next trial can start as predefined even if the audio file from the previous trial has not yet been transferred to the server. For anyone interested in more details of the technical background, we recommend consulting the MDN Web Docs site as a starting point, as this site provides information about Open Web technologies including JavaScript, HTML, CSS, and APIs (https://developer.mozilla.org/de/).

### Data analysis

#### Data preprocessing

For the *SoSciSurvey* data, the recordings were first converted to wav format from the browser’s default compressed recording format. For the jsPsych data, the recordings were already in wav format. To extract the naming latency from the audio recordings in the overt naming task, all audio files were then processed with the tool *Chronset *(Roux et al., 2017)*,* which is an automated tool for the detection of speech onsets from audio files. Afterwards, the audio files were manually checked using the software *Praat *(Boersma & Weenink, 2020) and a custom script (van Scherpenberg et al., 2020) to ensure that participants were producing the correct target word and to manually correct the determined speech onset where necessary.

#### Data exclusion

##### Replacement of participants due to prescreening of data

**Fig. 1:**
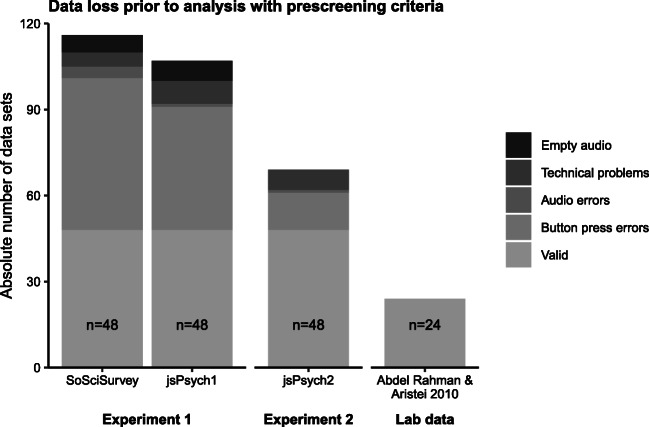
The number of individual data sets that had to be collected in order to obtain the pre-defined sample size and the number of data sets that was excluded based on our preregistered inclusion criteria in Experiment 1 and 2. For comparison, the figure also depicts the lab-based experiment from Abdel Rahman & Aristei (2010). In that study, no data sets had to be removed

Participants were excluded and replaced in the dataset if more than 20 % of the trials were incomplete or marked as deficient. Trials were marked as deficient if (1) participants produced an error (wrong picture name or wrong letter classification ), (2) the audio files of the naming response did not contain any sound, or (3) if there were other technical difficulties concerning the audio files. These difficulties included excessive background noise, an extremely low audio signal, or irregular lengths of the audio file within a participant. In the *SoSciSurvey* version of the experiment, irregular file lengths were so pervasive that we did not consider it practical to replace participants on these grounds in this version. See the Discussion for details on this issue. See also Fig. 1 for an overview of the exclusion of participants due to the prescreening criteria.

##### Data exclusion of single trials

In the data of the final 48 participants in both versions of the experiment, single trials were excluded if no response was given, participants made an erroneous response, or if participants responded prematurely (i.e., reaction times under 200 ms). See Table 1 for an overview of the data exclusion of single trials.

**Table 1:** Data loss caused by preprocessing the final samples of *n* = 48 in % of total data in Experiment 1 (*SoSciSurvey* and *jsPsych1*) and Experiment 2 (*jsPsych2*). Trials were excluded from analysis if participants did not press a button in the binary button-press classification task (button-press task – no reaction), classified the last letter incorrectly (button-press task – error), did not produce an object name in the naming task (naming task – no reaction), did not produce the correct target word in the naming task (naming task – error), or if a voice onset of less than 200 ms was registered (naming task – early response)

Exclusion due to	Experiment 1		Experiment 2
	SoSciSurvey	jsPsych1	jsPsych2
button-press task – no reaction	1.89	1.48	2.12
button-press task – error	3.79	3.92	2.90
naming task – no reaction	0.44	0.78	0.20
naming task – error	0.69	2.00	1.39
naming task – early response	0.12	0.72	0.17
data loss	6.93	8.91	6.78

#### Data transformation and selection of linear mixed effect models

To approximate a normal distribution of the residuals of the dependent variable, the Box–Cox power transformation procedure was applied to the response latency data (Box & Cox, 1964). The specific transformation that was performed is noted in the respective section of the Results.

For analysis of the response latency data, we used the packages *lme4*(Bates, Mächler, et al., 2015b) and lmerTest (Kuznetsova et al., 2017) in the statistical software R (R Core Team, 2019; Version: 3.6.1) to fit linear mixed effect models (LMMs) of the (transformed) response latency with the fixed effect predictors *task (button-press vs. overt naming task)*, *relatedness (related vs. unrelated)*, as well as their interaction. Both predictors were coded as sum contrasts (*button-press – overt naming* and *related – unrelated*, respectively). In order to examine more closely the effect of relatedness in the two tasks, nested LMMs were also fitted, in which the fixed effect of *relatedness* was estimated separately for the two levels of the factor *task*. The additional factors *repetition* (whether a picture was seen for the first or second time within a task) and *task-order* (button-press—naming vs. naming—button-press) were included as separate fixed effects (both contrast-coded). If their inclusion led to an increase in model fit, as indicated by a likelihood ratio test, they remained in the final model.

In specifying the structure of the models’ random effects, we followed the procedure outlined by Bates and colleagues (2015a). Initially, a full model with the complete variance-covariance matrix of the random effects allowed for by the design (i.e., random effects by subject and by picture) was fitted. This model was then simplified by first forcing the correlation parameters between the random effects to zero, then identifying overfitting of the parameters in the random effects using principal component analysis and dropping those random effects that contributed the least to the cumulative proportion of variance as identified by the principal component analysis until dropping a random effect led to a reduction in the goodness of fit. Correlation parameters between random effects were then reintroduced and kept in the final model if their re-inclusion led to an increase in the model fit and did not lead to non-convergence of the model. Models reported in the Results section are always final, reduced models.

### Results

**Fig. 2:**
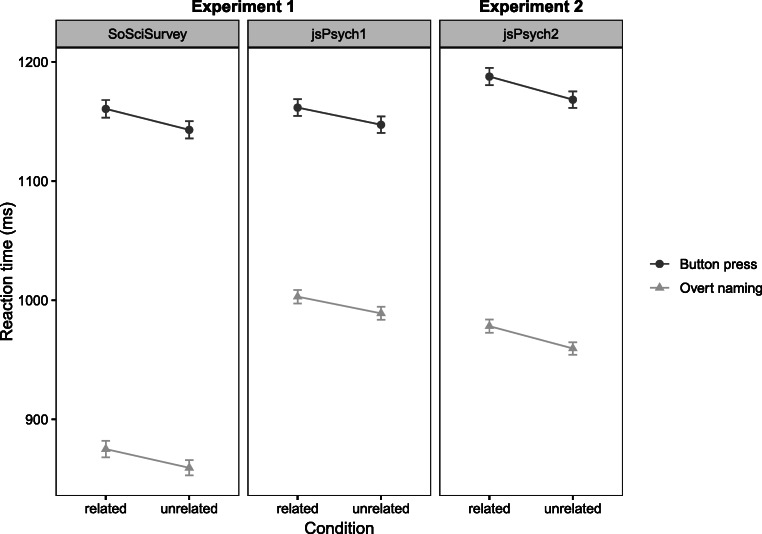
Mean reaction times in ms with standard error of means for naming and button-press tasks in both implementations of the online PWI in Experiment 1 (*SoSciSurvey* and *jsPsych1*) and Experiment 2 (*jsPsych2*). Targets presented with a semantically related distractor were classified and named slower than targets with unrelated distractors

**Fig. 3:**
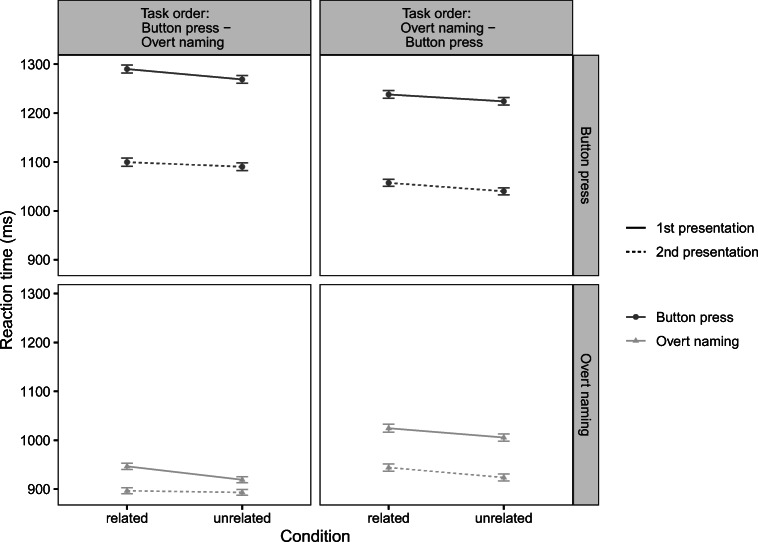
Mean reaction times in ms with standard error of means with pooled data from all online experiments plotted separately for task and task sequence. The figure can be read columnwise from top to bottom for comparing the effect of picture repetition within one task sequence. The *left column* represents the task sequence 1st button-press trials – 2nd overt naming trials and the right column depicts the task sequence 1st overt naming trials – 2nd button-press trials. Furthermore, the figure can be read rowwise from left to right in the upper row for comparing the effect of picture repetition (1st to 4th) within the button-press task and rowwise from right to left in the lower row for comparing the effect of picture repetition (1st to 4th) within the overt naming task

**Fig. 4:**
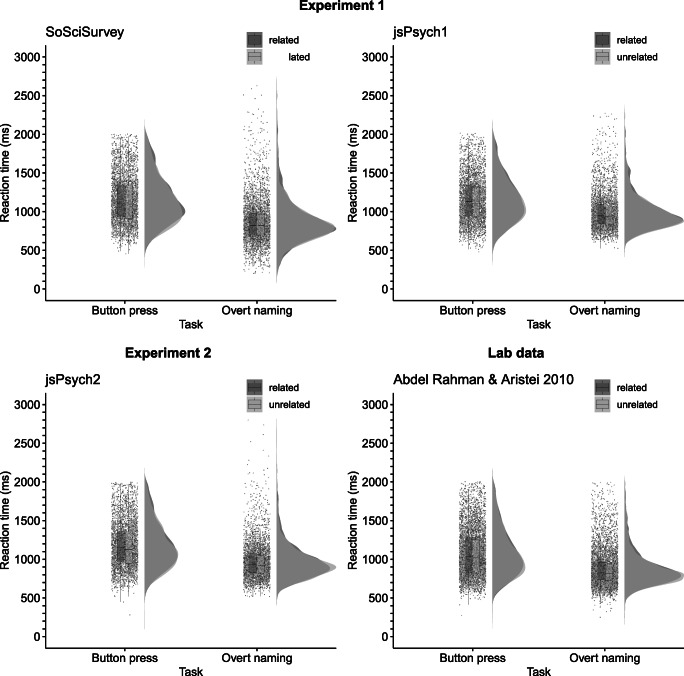
Single trial plots (before model criticism) for the factors task and relatedness in all three experiments and the lab-based study by Abdel Rahman & Aristei. *Box plots* represent the median per relatedness condition with lower and upper hinges corresponding to the 25th and 75th percentiles and *whiskers* extending to the most extreme value within 1.5*IQR from the box hinges

In the *SoSciSurvey* data, the mean response latency in the button-press task was 1161 ms (SE = 7 ms) in the related condition and 1143 ms (SE = 7 ms) in the unrelated condition. In the overt naming task, the mean response latency was 875 ms (SE = 7 ms) in the related condition and 859 ms (SE = 6 ms) in the unrelated condition. In the *jsPsych* data, the mean response latency in the button-press task, was 1162 ms (SE = 7 ms) in the related condition and 1147 ms (SE = 6 ms) in the unrelated condition. In the overt naming task, the mean response latency was 1003 ms (*SE* = 6 ms) in the related condition and 989 ms (SE = 5 ms) in the unrelated condition. See Fig. 2 for line plots of mean response latency by *task* and *relatedness* for both versions of the experiment, Fig. 3 for line plots of mean response latency by task, relatedness and repetition for all online experiments and Fig. 4 for raincloud plots of the single trial data and their distribution by *task* and *relatedness* for all experiments as well as for the previous study by Abdel Rahman and Aristei (2010). Note that overall response latencies are slower in the online experiments compared to the lab. We discuss possible reasons for this in the General discussion section.

#### Preregistered analysis

##### SoSciSurvey

For the *SoSciSurvey* data, the box-cox procedure suggested a log-transformation of the response latency variable. In the final model (containing main effects of *task, relatedness,* and their interaction) both main effects were significant, while the interaction task*relatedness was not significant. The positive sign of the estimate for *task* and the coding of the *task* contrast show that response latency was slower in the button-press task compared to the naming task. Likewise, for *relatedness* the response latency was slower in the related than in the unrelated condition. In the final nested model (i.e. estimating separate fixed effects of *relatedness*, for the two levels of task), the nested effect of *relatedness* was marginally significant in the button-press task and did not reach significance in the overt naming task. See Table 2 for an overview of the reported models from the preregistered analysis including model formula, coefficients and random effect variance parameters.

**Table 2:** Table of final models from the preregistered analysis of the *SoSciSurvey* version of Experiment 1 (*SoSciSurvey* and *jsPsych1*). Indexing of estimate column denotes which transformation was applied to the dependent variable. *** = p < .001; ** = p < .01: * = p < .05

Model	Formula			
*SoSciSurvey* full model, no outlier correction	log(rt) ~ 1 + task + relatedness + repetition + task:relatedness + (1 + task | subject) + (1 + task + relatedness | picture)			
Fixed effects	Estimate_log_	Std. Error	*t* value	*p* value
Intercept	6.87	0.02	316.99	< .001***
Task	0.3	0.03	10.09	< .001***
Relatedness	0.01	0.01	2.06	.046*
Repetition	0.13	0.01	23.96	< .001***
Task x Relatedness	– 0.001	0.01	-0.09	.93
Random effects	Variance	Std. Deviation		
Subjects				
Intercept	0.02	0.13		
Task	0.04	0.19		
Pictures				
Intercept	0.005	0.07		
Task	0.003	0.05		
Relatedness	0.001	0.03		
Residual	0.05	0.22		
*Goodness of fit*				
Log likelihood	371.4			
Model	Formula			
*SoSciSurvey* nested, no outlier correction	log(rt) ~ 1 + task/relatedness + repetition + (1 + task | subject) + (1 + task + relatedness_naming_ | picture)			
Fixed effects	Estimate_log_	Std. Error	*t*-value	*p*-value
Intercept	6.87	0.02	317.09	< .001***
Task	0.3	0.03	10.08	< .001***
Relatedness_BP_	0.01	0.01	1.80	.07
Relatedness_naming_	0.02	0.01	1.50	.14
Repetition	0.12	0.01	23.95	< .001***
Random effects	Variance	Std. Deviation		
Subjects				
Intercept	0.02	0.13		
Task	0.04	0.19		
Pictures				
Intercept	0.005	0.07		
Task	0.003	0.05		
Relatedness_naming_	0.002	0.05		
Residual	0.05	0.22		
*Goodness of fit*				
Log likelihood	375.7			

##### jsPsych1

For *jsPsych1*, the Box–Cox procedure suggested transforming the response latency variable by raising to the power of – 0.5 (i.e., 1 divided by the square root of the variable). This type of transformation reverses the sign of a model’s parameter estimates compared to log-transformed or untransformed data. We therefore transformed using – 1 in the nominator (– 1/square root of the variable) to maintain the same sign as in the other (log-transformed) models. In the final model, both main effects *task* and *relatedness* were significant, while their interaction was not significant. In the final nested model, the nested effect of *relatedness* was significant in the button-press task and marginally significant in the overt naming task. See Table 3 for an overview of the reported models from the preregistered analysis including model formula, coefficients and random effect variance parameters.

**Table 3:** Table of final models from the preregistered analysis of the *jsPsych1* version of Experiment 1 (*SoSciSurvey* and *jsPsych1*). Indexing of estimate column denotes which transformation was applied to the dependent variable. *** = p < .001; ** = p < .01: * = p < .05

Model	Formula			
*jsPsych1* full model, no outlier correction	-1/sqrt(rt) ~ 1 + task + relatedness + repetition + task:relatedness + (1 + task || subject) + (1 + task + relatedness || picture)			
Fixed effects	Estimate_-1/sqrt_	Std. Error	*t*-value	*p*-value
Intercept	-0.03	0.0003	119.44	< .001***
Task	0.002	0.0003	6.68	< .001***
Relatedness	0.0002	0.0001	2.23	.031*
Repetition	0.002	0.0001	23.59	< .001***
Task x Relatedness	0.00004	0.0001	0.28	.77
Random effects	Variance	Std. Deviation		
Subjects				
Intercept	0.000002	0.001		
Task	0.000004	0.002		
Pictures				
Intercept	0.0000009	0.001		
Task	0.0000005	0.001		
Relatedness	0.0000002	0.0004		
Residual	0.000008	0.003		
*Goodness of fit*				
Log likelihood	30889.8			
Model	Formula			
*jsPsych1* nested, no outlier correction	-1/sqrt(rt) ~ 1 + task/relatedness + repetition + (1 + task || subject) + (1 + task + relatedness_naming_ || picture)			
Fixed effects	Estimate_-1/sqrt_	Std. Error	*t*-value	*p* value
Intercept	-0.03	0.0003	119.53	< .001***
Task	0.002	0.0003	6.68	< .001***
Relatedness_BP_	0.0002	0.0001	2.07	.038*
Relatedness_naming_	0.0002	0.0001	1.77	.08
Repetition	0.002	0.0001	23.59	< .001***
Random effects	Variance	Std. Deviation		
Subjects				
Intercept	0.000002	0.001		
Task	0.000004	0.002		
Pictures				
Intercept	0.0000009	0.001		
Task	0.0000005	0.001		
Relatedness_naming_	0.0000003	0.001		
Residual	0.000008	0.003		
*Goodness of fit*				
Log likelihood	30888.8			

#### Exploratory analyses

While in both versions of the experiment the *relatedness* effect was significant in the models containing the main effect of *relatedness* across tasks and did not interact with the factor *task*, *relatedness* did not reach significance when examining the effect separately for the overt naming task. It is plausible to assume that the overt naming data from an online environment would suffer from an increased level of noisiness and this is also evident in the longer tails of the response time data when comparing the single trial data from Experiment 1 to the data from Abdel Rahman and Aristei (2010), see Fig. 4. Therefore, we performed outlier correction employing an approach specifically tailored to LMMs suggested by Baayen and Milin (2010). This approach relies on model criticism after model fitting rather than a priori screening for extreme values, by removing those data points with absolute standardized residuals that exceed 2.5 standard deviations. Baayen and Milin demonstrated that this approach proves more conservative (i.e., excludes fewer data points) compared to more traditional approaches to outlier correction. For the nested models, this led to the exclusion of 2.39% of trials for the prescreened *SoSciSurvey* data and 1.75% of trials for the prescreened *jsPsych1* data.

Refitting the final nested model with the outlier corrected *SoSciSurvey* data, the nested effect of *relatedness* was significant in the button-press task but not significant in the overt naming task. In the refitted final model of the outlier corrected *jsPsych1* data, the nested effect of *relatedness* was significant in the button-press task and in the overt naming task. See Table 4 for an overview of the reported models from the preregistered analysis including model formula, coefficients, and random effect variance parameters.

**Table 4:** Table of final models from the exploratory analysis of outlier corrected data from Experiment 1 (*SoSciSurvey* and *jsPsych1*). Indexing of estimate column denotes which transformation was applied to the dependent variable. *** = p < .001; ** = p < .01: * = p < .05

Model	Formula			
*SoSciSurvey* nested, with outlier correction	log(rt) ~ 1 + task/relatedness + repetition + (1 + task | subject) + (1 + task + relatedness_naming_ | picture)			
Fixed effects	Estimate_log_	Std. Error	*t*-value	*p*-value
Intercept	6.86	0.02	317.58	< .001***
Task	0.3	0.03	10.29	< .001***
Relatedness_BP_	0.01	0.01	2.17	.03*
Relatedness_naming_	0.01	0.01	1.1	.28
Repetition	0.13	0.01	27.15	< .001***
Random effects	Variance	Std. Deviation		
Subjects				
Intercept	0.02	0.13		
Task	0.04	0.2		
Pictures				
Intercept	0.005	0.07		
Task	0.003	0.05		
Relatedness_naming_	0.002	0.05		
Residual	0.04	0.2		
*Goodness of fit*				
Log likelihood	1140.5			
Model	Formula			
*jsPsych1* nested, with outlier correction	-1/sqrt(rt) ~ 1 + task/relatedness + repetition + (1 + task || subject) + (1 + task + relatedness_naming_ || picture)			
Fixed effects	Estimate_-1/sqrt_	Std. Error	*t*-value	*p*-value
Intercept	– 0.03	0.0003	118.11	< .001***
Task	0.002	0.0003	6.86	< .001***
Relatedness_BP_	0.0002	0.0001	2.17	.03*
Relatedness_naming_	0.0003	0.0001	2.07	.045*
Repetition	0.002	0.0001	24.36	< .001***
Random effects	Variance	Std. Deviation		
Subjects				
Intercept	0.000002	0.001		
Task	0.000004	0.002		
Pictures				
Intercept	0.0000009	0.001		
Task	0.0000006	0.001		
Relatedness_naming_	0.0000004	0.001		
Residual	0.000007	0.003		
*Goodness of fit*				
Log likelihood	30854.8			

### Discussion

The results of the two versions of the first experiment were promising regarding the demonstration of the semantic interference effect in an online setting.For both versions, we found a significant effect of *relatedness*, with slower response latencies when a picture was accompanied by a semantically related written distractor word compared to an unrelated distractor, replicating the classic picture–word interference effect observed in lab settings. As we did not find an interaction of the effect of *relatedness* with the factor *task*, this effect appears to be independent of the task.

As the particular focus of the current study was demonstrating that voice onset latencies could be collected in online settings, we examined the two tasks separately to investigate the effect of *relatedness* specifically in the overt naming task. When looking at the effect in both tasks separately via nested models, we found that the effect did not reach significance in the overt naming task when we ran the preregistered analyses without any outlier correction. When we applied outlier correction by using model criticism and ran the models on the corrected data, the *relatedness* effect in the overt naming task reached significance in the *jsPsych1* version, but not in the *SoSciSurvey* version (where the *t* value of the estimate actually decreased). An additional model of the overt naming data from both experimental platforms did not yield a significant interaction of the factors platform (*SoSciSurvey* vs. *jsPsych*) and *relatedness* (*b* = 0.001, *t* = 0.153, *p* = 0.878). The absence of this interaction indicates that it is not possible to conclude that the relatedness effect is actually stronger in one platform compared to the other. Even so, the significance of the effect depended on an outlier correction which, while specifically tailored to single trial data in the context of LMMs, we had not planned and therefore not preregistered. It is very likely and plausible that response latency measurements collected via the audio recordings employed in our online experiments suffer from increased random error, i.e. noise, which would decrease the power to find effects.

Presumably, one of the factors contributing to this increased noisiness of the data is the technical implementation of the audio recordings and the reliability of the recordings’ timing relative to stimulus onset. This is reflected, for example, in the issue of the variability of the audio file lengths. Only very few of the participants’ audio files were exactly of the anticipated length of 3000 ms. Presumably, deviations from this file length are due to factors like audio sampling rate, technical variability between the users’ machines, and fluctuation in Internet connection quality. It is beyond the scope and goal of this study to address the technical details of the recording process and to solve the problems underlying the variable file lengths. As long as the file lengths for a single participant were homogeneous, we expected the recording process for that participant to be reliable enough to determine reliable speech onsets. Importantly, in the majority of datasets within the *jsPsych1* version, the file lengths were homogeneous *within* any one participant, and only a few participants (ten out of 59 participants) showed considerable variability of file lengths (i.e., in more than 20% of files). We excluded and replaced these ten participants, as we could not rule out that in shorter audio files the recording started later than programmed and thus we might not be able to infer the correct voice onset timing.

In the *SoSciSurvey* audio files, the issue was a lot more pervasive. In contrast to the *jsPsych1* data set, 31 of 48 participants included in the final dataset of the *SoSciSurvey* version showed within-subject variability of file lengths in more than 20% of files. The issue was present more frequently than absent, which meant that excluding and replacing these participants would have further escalated the already large number of participants required to be collected before reaching the target sample size. The implementation of audio recordings employed in *SoSciSurvey* would therefore seem to be more susceptible to the variability within any one participant’s technical setup. If the variability of the audio file length is an indication of the reliability of the timing of the audio recording within the experiment, the measurements in jsPsych are more reliable.

An additional issue concerning the reliability of the timing of audio recordings in both platforms is the overall difference between the response latencies in the overt naming task of the two versions: voice onset latencies were quicker in *SoSciSurvey* compared to *jsPsych1* (M = 867 ms across *relatedness* for *SoSciSurvey* vs. M = 996 ms for *jsPsych1*). We cannot account for this difference, as the two versions of the experiment were nearly identical and therefore interpret it as a technical issue in the audio recording implementation of the *SoSciSurvey* version. This would align with the finding that the *relatedness* effect in the jsPsych version could also be found when looking at the overt naming task separately in the nested model after exclusion of outliers with a model criticism procedure.

Nevertheless, even the *jsPsych1* version had its difficulties and the effect in the overt naming task was only marginally significant in our preregistered analysis. To assess if the effect in this version was a stable finding or a spurious result, we decided to conduct a second experiment using the same implementation with jsPsych as experiment builder and JATOS as server with several minor adjustments.

One apparent problem with both versions of the first experiment was the high number of data sets which had to be excluded according to our preregistered inclusion criteria. The majority of the excluded participants made too many errors in the button-press task, often erroneously classifying the written distractor words’ last letter instead of the targets’. Adjusting the instructions in the follow-up experiment to be clearer with respect to the button-press task, and providing examples of correct responses to practice trials should improve the error rates and therefore make data collection more efficient. In another adjustment to address the problem of participant exclusion rates, we decided to recruit the participants for the second experiment via the institute’s participant pool instead of Prolific, as these participants may be more accustomed to reaction time experiments similar to the current study.

Furthermore, collecting a second dataset using the jsPsych experiment builder would also allow to pool both datasets in a separate analysis, thereby increasing the power to find an effect.

## Experiment 2

Experiment 2 was separately preregistered on AsPredicted.com (AsPredicted#: 49281, available at https://aspredicted.org/blind.php?x=9q2yf3).

### Methods

The second experiment *jsPsych2* was identical to *jsPsych1* with the exception of a few changes.

#### Participants

In total, 69 native German speakers were recruited using the institutes’ participant pool Psychologischer Experimental Server Adlershof (PESA). They were included in the final sample when meeting all exclusion criteria until the final sample consisted of 48 participants as determined in the preregistration (36 females, 18–35 years, *M M*_age_ = 23.69, _*age*_  SD_age_ = 4.99). Participants provided informed consent to their participation in the study. The study was conducted based on the principles expressed in the Declaration of Helsinki and was approved by the local Ethics Committee. Participants received course credit or a monetary compensation.

#### Procedure

To increase the efficiency of the data collection and to decrease the high error rates in the first two versions of Experiment 1, an explicit instruction to ignore the written distractor words was included. In addition, each practice trial was followed by an example of what the correct response should have been.

#### Data exclusion

Participants were excluded and replaced based on the same criteria as in Experiment 1, see Fig. 1 for an overview. Similar to the *jsPsych* version of Experiment 1, the issue of irregular file lengths only occurred in a few participants (six of 69), which were excluded and replaced. In the data set of the final 48 participants, trials were excluded if no response was given, participants made an erroneous response, or if participants responded prematurely. See Table 1 for an overview of the data exclusion of single trials. For Experiment 2, outlier correction via model criticism was applied from the beginning and the models reported in the Results section were fitted to the outlier corrected data. A further 1.67% of trials were excluded following the model criticism procedure.

### Results

#### Preregistered analysis

In the button-press task, the mean response latency was 1188 ms (SE = 7 ms) in the related condition and 1168 ms (SE = 7 ms) in the unrelated condition. In the overt naming task, the mean response latency was 978 ms (SE = 6 ms) in the related condition and 960 ms (SE = 6 ms) in the unrelated condition. See Fig. 2 for a depiction of the impact of *task* and *relatedness* on response latencies for all three versions of the experiment and see Fig. 4 for raincloud plots of the single trial data and their distribution by task and relatedness for all experiments as well as for the previous study by Abdel Rahman and Aristei (2010).

The Box–Cox procedure suggested the same transformation as for the *jsPsych1* data: – 1 divided by the square root of the response latency variable. In the final model of the outlier corrected data, both main effects *task* and *relatedness* were significant, while there was no significant interaction. In the final nested model, the nested effect of *relatedness* was significant in the button-press task and in the overt naming task. This indicates that it takes longer to classify and name a target picture if it is presented together with a semantically related distractor. See Table 5 for an overview of the reported models of Experiment 2 including model formula, coefficients and random effect variance parameters.

**Table 5:** Table of final models from the preregistered analysis of Experiment 2 (*jsPsych2*). Indexing of estimate column denotes which transformation was applied to the dependent variable. *** = p < .001; ** = p < .01: * = p < .05

Model	Formula			
*jsPsych2* full model, with outlier correction	-1/sqrt(rt) ~ 1 + task + relatedness + repetition + task:relatedness + (1 + task || subject) + (1 + task + relatedness || picture)			
Fixed effects	Estimate_-1/sqrt_	Std. Error	*t*-value	*p* value
Intercept	– 0.03	0.0003	118.44	< .001***
Task	0.003	0.0003	10.89	< .001***
Relatedness	0.0002	0.0001	2.73	< .01**
Repetition	0.002	0.0001	28.78	< .001***
Task x Relatedness	0.00003	0.0001	0.24	.81
Random effects	Variance	Std. Deviation		
Subjects				
Intercept	0.000002	0.001		
Task	0.000003	0.002		
Pictures				
Intercept	0.0000009	0.001		
Task	0.0000004	0.001		
Relatedness	0.0000002	0.0004		
Residual	0.000007	0.003		
*Goodness of fit*				
Log likelihood	31715.2			
Model	Formula			
*jsPsych2* nested, with outlier correction	– 1/sqrt(rt) ~ 1 + task/relatedness + repetition + (1 + task || subject) + (1 + task + relatedness_naming_ || picture)			
Fixed effects	Estimate_-1/sqrt_	Std. Error	*p*-value	*p*-value
Intercept	– 0.03	0.0003	118.47	< .001***
Task	0.003	0.0003	10.84	< .001***
Relatedness_BP_	0.0002	0.0001	2.67	.008**
Relatedness_naming_	0.0003	0.0001	2.18	.035*
Repetition	0.002	0.0001	29.02	< .001***
Random effects	Variance	Std. Deviation		
Subjects				
Intercept	0.000002	0.001		
Task	0.000003	0.002		
Pictures				
Intercept	0.0000009	0.001		
Task	0.0000004	0.001		
Relatedness_naming_	0.0000003	0.001		
Residual	0.000006	0.003		
*Goodness of fit*				
Log likelihood	31702.3			

### Discussion

The results of Experiment2 (*jsPsych2*) confirmed the results from the *jsPsych1* version of Experiment 1. The effect of relatedness was significant in the full model as well as in both tasks separately in the nested model, replicating the semantic interference effect in the PWI in general, and the findings from Abdel Rahman and Aristei (2010) in particular. The implementation of audio recordings in the online environment offered by jsPsych and JATOS seems to be able to provide stable measurements of verbal response latencies.

The adjustments to the procedure in *jsPsych2* also improved the efficiency of the online data collection. Whereas in *jsPsych1,* datasets from a total of 108 participants needed to be collected to reach the desired sample size of 48, only 69 participants were required in experiment *jsPsych2* to reach the same goal. While the main reason for exclusion was still participants’ error rate in the button-press task, the number of participants with an error rate above 20% decreased from 43 participants in *jsPsych1* to 13 participants in *jsPsych2*. Furthermore, most of these 13 participants had an error rate only slightly above our predefined threshold indicating that they did not misunderstand the task and classified the distractor instead of the target, which had been the case for *jsPsych1*.

### Pooled analysis and post hoc analyses of power

To increase the power of the analysis, the data from *jsPsych1* and *jsPsych2* was pooled. The model criticism procedure for the pooled data resulted in the exclusion of an additional 1.71% of trials, compared to the pooled data of the two experiments without any outlier correction.

The Box–Cox procedure suggested the same transformation as for the *jsPsych1* and the *jsPsych2* data: – 1 divided by the square root of the response latency variable. In the final model, both main effects of *task* and *relatedness* were significant, in the absence of an interaction. In the final nested model, the nested main effect of *relatedness* was significant in the button-press task and in the overt naming task. See Table 6 for an overview of the reported models from the pooled analysis including model formula, coefficients and random effect variance parameters.

**Table 6:** Table of final models from the preregistered analysis of the pooled analysis (*jsPsych1 +jsPsych2*). Indexing of estimate column denotes which transformation was applied to the dependent variable. *** = *p* < .001; ** = *p* < .01: * = *p* < .05

Model	Formula			
*Pooled (jsPsych1 + jsPsych2)* full model, with outlier correction	– 1/sqrt(rt) ~ 1 + task + relatedness + repetition + task:relatedness + (1 + task || subject) + (1 + task + relatedness || picture) + (0 + task || experiment)			
Fixed effects	Estimate_log_	Std. Error	*t*-value	*p* value
Intercept	– 0.03	0.0002	144.97	< .001***
Task	0.003	0.0003	7.83	.007**
Relatedness	0.0002	0.0001	2.45	.008**
Repetition	0.002	0.00004	35.24	< .001***
Task x Relatedness	0.00006	0.0001	0.75	.49
Random effects	Variance	Std. Deviation		
Subjects				
Intercept	0.000002	0.001		
Task	0.000004	0.002		
Pictures				
Intercept	0.000001	0.001		
Task	0.0000005	0.001		
Relatedness	0.0000002	0.001		
Experiment				
Task	0.0000001	0.0003		
Residual	0.000007	0.003		
*Goodness of fit*				
Log likelihood	62624.9			
Model	Formula			
*Pooled (jsPsych1 + jsPsych2)* nested, with outlier correction	– 1/sqrt(rt) ~ 1 + task/relatedness + repetition + (1 + task || subject) + (1 + task + relatedness_naming_ || picture)			
Fixed effects	Estimate_-1/sqrt_	Std. Error	*t*-value	*p*-value
Intercept	-0.03	0.0003	145.03	< .001***
Task	0.003	0.0003	7.84	.01*
Relatedness_BP_	0.0002	0.0001	2.22	.008**
Relatedness_naming_	0.0003	0.0001	2.1	.02*
Repetition	0.002	0.0001	35.24	< .001***
Random effects	Variance	Std. Deviation		
Subjects				
Intercept	0.000002	0.001		
Task	0.000004	0.002		
Pictures				
Intercept	0.000001	0.001		
Task	0.0000005	0.001		
Relatedness_BP_	0.0000001	0.0002		
Relatedness_naming_	0.0000003	0.001		
Experiment				
Task	0.0000001	0.0003		
Residual	0.000007	0.003		
*Goodness of fit*				
Log likelihood	62624.9			

As expected, the effect of *relatedness* in both tasks was more stable when pooling the data from two experiments and thus increasing the power of the analysis. To determine to what extent this pooled analysis might have been ‘overpowered’ and to find a balance between sufficient power on the one hand and sensible sample sizes on the other hand, we performed a post hoc power analysis with the package *simr*(Green & MacLeod, 2016) using the parameter estimates derived from a separate model of only the overt naming task from the pooled data.^2^

**Fig. 5:**
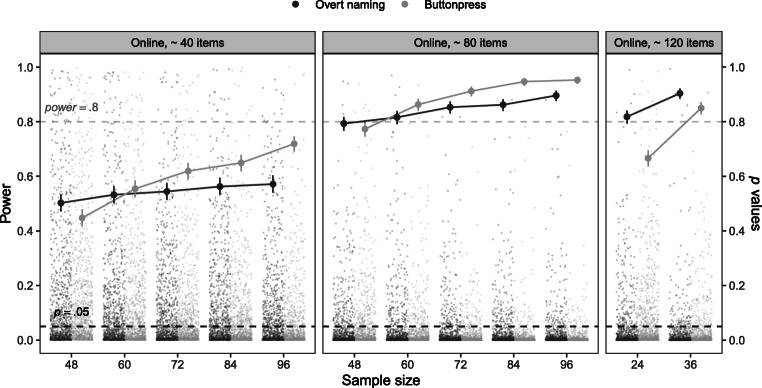
Results of the post hoc power simulations for the fixed effect of *relatedness* in both tasks based on estimates from the pooled analysis with an increase in both sample size (on the *x*-axis) and number of items (different panels). The *big dots* represent power plotted by different sample sizes. For each sample size, the number of simulations to estimate power was *n* = 1000. The *small dots* represent the resulting *p* values for each of the 1000 simulations. Increases in power result from higher proportions of runs with *p* values below the threshold of *p* = .05. The *dashed grey line* represents the threshold for reaching a power of 80%.

For the post hoc power analysis, we calculated a power curve, which relies on 1000 simulated data sets based on parameters from our pooled data set. These simulated data sets were then analyzed with the proportion of significant results relative to all simulations indicating the respective power (Kumle, Võ, & Draschkow, 2021). Figure 5 displays the estimated power for increasing sample sizes and increasing number of trials.

The observed power to find a significant effect of *relatedness* increased with growing sample sizes as expected. At a sample size of 96 (i.e., the actual sample size of the pooled analysis) and 40 trials as in this experiment the simulated power is 72% for the button-press task and 60% for the overt naming task. These values are notably lower than the 80% power with a sample size of 36 participants that we determined via an a priori power analysis (see Methods section of Experiment 1). A possible reason for these differences is the fact that for the a priori analysis we relied on estimates from a lab-based study and only included random intercepts, whereas the model estimates used in the post hoc analysis included a random slope parameter for item as well as a correlation parameter of the random intercept and slope by item, as determined empirically by our model selection process. This resulted in an increase in the number of parameters between the models from four in the a priori analysis to six in the post hoc analysis. Matuschek et al. (2017) point out that fitting more complex models with more random effect parameters comes at a cost of power. Furthermore, it is notable that the increase in power with increasing sample sizes is not very large, especially for the overt naming task. However, increasing the number of items seems to be more beneficial and important than increasing the observations within each subject in order to reach an estimated power of 80%. Indeed, when running simulations increasing both sample size and the number of items, we see that the power strongly increases from 40 to 80 items. For example, a study with 60 participants and 80 items or with 36 participants and 120 trials would yield an expected power of 80% . Thus, under some conditions, future experiments may profit more from an increase in items rather than an increase in subjects. However, see our recommendations in the General discussion for possible drawbacks to this approach.

## General discussion

In the present study, we introduced three online implementations of the PWI task and replicated the well-known semantic interference effect. To the best of our knowledge, this is the first time stimulus-locked voice recordings from an online experiment have been used to successfully measure voice onset latencies as a dependent variable, providing a proof of concept that language production experiments relying on overt naming can be moved online.

In our implementations of an online PWI task, we presented pictures with visually superimposed distractor words that were either semantically related or unrelated to the target picture. Participants were asked to name the picture (overt naming) or, as a control task, classify the last letter of the picture name as a vowel or consonant (button-press). Our goal was to find the typically observed semantic interference effect with longer response latencies if a distractor word and a picture are semantically related (vs. unrelated). Given the poor audiovisual synchrony reported for different web experiment builders, browsers, and hardware configurations (Bridges et al., 2020) in combination with the small size of the semantic interference effect of around 20 ms, this was not a trivial endeavor. Despite these challenges, we replicated the semantic interference effect in both tasks, classification and overt articulation in three pre-registered experiments, and the effect closely resembles in size the effect reported by Bürki et al. (2020) in their recent metastudy on semantic interference in the PWI task. We conclude that running language production experiments online is feasible.

### Data quality in our implementation of online language production experiments

#### Data loss

Comparing the amount of data loss over the course of the three experiments with studies run in the lab it is evident that a higher number of participants had to be tested in order to reach our predefined goal of collecting 48 valid data sets. In the first two runs of the study, more than double the number of data had to be collected in order to obtain at least 80% of trials per task and participant for analysis. The reasons for this high loss of data sets are two-fold.

One source of error are the participants themselves. We found that many participants did not read the instructions carefully enough and hence had a high error rate when classifying the last letters. This was especially pronounced in the first experiment where many participants classified the distractor word instead of the target. However, this problem was minimized in experiment *jsPsych2* where we used a participant pool that might be more accustomed to lab settings and by giving more explicit instructions as well as providing performance feedback by giving the correct response in the practice trials. Although the number of participants with an error rate above 20% was still non-negligible, the number of participants always classifying the distractor word was reduced substantially by these measures. The second reason for loss of data sets can be subsumed under technical problems. We encountered cases with empty audio files, differing file lengths within participants, noise on audio files, as well as files of poor audio quality. Note, that there were no empty audio files in Experiment 2, indicating that empty files might be a result from non-compliant participants muting their microphones (we presume our participants were more compliant in Experiment 2).

#### Noisiness of data

In our experience, data collected online was noisier than data collected in the lab with longer tails in the distribution of response time data compared to the lab experiment by Abdel Rahman and Aristei (2010) and longer overall response times. This has also been found by other groups running online language production studies recently (Fairs & Strijkers, 2021). We deem it likely that the lack of a controlled testing environment when testing online is the reason for these relatively long reaction times. However, as can be seen from Fig. 3, we do find classic repetition effects with participants getting faster with repeated stimulus presentations. This underlines our conclusions that online testing is a suitable approach for using language production paradigms relying on the estimation of voice onset latencies.

Still, even though we accounted for the potential greater noisiness of online data a priori by raising the number of participants by a third after running a power estimation for the effect from a previous lab experiment, the interference effect for naming was only marginally significant in Experiment *jsPsych1.* To counter noise in the data, we performed an outlier screening by applying model criticism. This improved the quality of the obtained data but has not been necessary to obtain interference effects in lab-based experiments. Furthermore, pooling data from both jsPsych experiments and thus increasing sample size made the interference effect more stable. Therefore, it seems likely that we underestimated the noisiness of online language production data in our first experiment.

#### Efficiency

In contrast to data collected in the lab using a voice key, the data generated online were not ready for analysis after their collection. First voice onset latencies were determined by using Chronset (Roux et al., 2017) and these were manually corrected as latencies were not computed reliably in all cases by this software tool. This procedure consumes time and resources, but could maybe be optimized in future.

In summary, contrary to other fields of behavioral psychology, running language production experiments online is not (yet) less resource consuming than research in the lab in order to obtain data sets of sufficiently high quality. We hope that future work might help to reduce these efforts. In the following, we provide recommendations based on our experiences that will likely improve the quality of the data.

### Recommendations for running online language production experiments

1. Take an informed decision on which web experiment builder you use

The choice of a web experiment builder may have a strong impact on the quality of the data in a web experiment (Bridges et al., 2020). Based on our data, we can strongly recommend using jsPsych (de Leeuw, 2015) for experiments in which audio responses are recorded – either implementing the audio-response-plugin which is provided in a beta version (Gilbert, 2020) or by using our custom script available on OSF (https://osf.io/uh5vr/?view_only=229679aa33604aa2a5cb400eab62099).

We tested different ways to implement online audio recordings using two web experiment builders for online experiments – SoSiSurvey (Leiner, 2019) and jsPsych (de Leeuw, 2015). Overall, we encountered less technical problems for jsPsych in comparison with the other experiment builder. Less audio files had to be discarded due to low quality and thus data loss was minimized to a substantial degree using jsPsych in combination with a custom audio-record plugin. We therefore advocate the use of jsPsych, a non-commercial and open-source software library for building web-based experiments with a proven record in a wide range of behavioral experiments. Many experimental tasks can easily be built by using the experimental plugins provided and even with little experience in JavaScript programming there are almost infinite possibilities to fine-tune them to cater to the needs of the experimenter. Furthermore, there is a very active and committed helper community available for questions that might arise during the process of developing the experiment. Presumably, using an audio recording option provided by other web experiment builders (e.g., LabVanced, Gorilla, Finding Five) would have led to similar results. However, no recording options for these experiment builders were available when preparing this study and a thorough examination of their timing reliability is still outstanding.

#### Custom audio recording implementation in jsPsych

As none of the available web experiment builders offered an audio recording option ensuring that audio recordings would be precisely time-locked to other stimuli, we customized a jsPsych plugin to our needs. With this plugin the experiment proceeds in the following way: When starting the experimental block, participants have to grant the browser access to their microphones once and microphone access remains active over the whole naming part. Within the naming part, the custom audio record plugin enables recording of short audio files of a predefined duration (in our case, 3 s) timelocked to the presentation of another stimulus (in our case a picture). The experiment then proceeds as defined in the experiment timeline, e.g., by presenting a fixation cross or the next trial. In the background, the audio files are transferred to the server where the experiment is hosted. We chose to transfer the files to a server using JATOS (Lange et al., 2015), an open-source tool for running online studies on your own server. Furthermore, JATOS also offers the option to structure the experiment in different components that are executed one after the other – a feature that we used in order to only have microphone access enabled in the naming part of the experiment but not during the following or preceding button-press task. However, the custom plugin should in principle be compatible with any other server that might best serve a researcher’s needs when hosting the experiment and saving the data. After the experiment, the audio files and log files can be downloaded and saved. Voice onset latencies can be extracted from the audio files and the onset latencies can be merged with the logfile from the experiment.

2. Limit noise stemming from technical side

It is essential to limit potential sources of noise stemming from the technical side. Noise can be introduced by variation in participants’ choice of browsers, their specific hardware and software. While it is not possible to have full control over the technical equipment of participants in an online experiment, it is possible to account for some of their potential influence. For example, we monitored which browser participants used in order to control whether data collected in one browser might be more or less reliable. We did not find evidence for specific browser-related differences in the reaction time data in the jsPsych experiment. However, during pretesting we found that the experiment was not working for Edge and sometimes not for Safari users. Therefore, we advised participants to run the experiment on a personal computer using Firefox or Chrome, thereby also minimizing the potential influence of different devices and browser types.

Furthermore, we strongly suggest to only use within-participant designs, which may reduce variability due to participants’ hardware and software setups and can help to minimize the influence of audiovisual synchrony problems on the experimental manipulation.

While future work on web experiment builders might help to reduce these technical problems, we deem it important to thoroughly screen the data gained from a web-based language production experiment and in case of doubt, rather exclude participants than keep participants with deficient data. To provide transparency to these decisions, we suggest preregistering sample size and data-exclusion criteria.

3. Limit noise stemming from participants

Samples reached via online testing may not be as cooperative and accustomed to the prerequisites of experiments as the standard lab population. Therefore, it is also essential to limit potential noise stemming from participants. For example, noise can be reduced by thoroughly instructing participants using catch questions to test their understanding of the task (Oppenheimer et al., 2009) and by giving ample feedback during practice trials. Furthermore, the pool from which participants are recruited may have an impact on data quality, as evident by the reduced number of data sets that had to be excluded in Experiment 2 for which we recruited participants from our institute’s subject pool.

Note that screening procedures and sampling strategies may ensure higher data quality while potentially minimizing the advantage of testing more diverse samples than in the lab. The chosen selection criteria may induce a sampling bias as e.g., stable Internet connections, well-set hardware, or the willingness to grant access to a microphone are not equally distributed. Therefore, researchers should be aware of the fact that online testing does not entail more representative samples than typical samples in the lab per se. Researchers should aim for a balance between high data quality and minimal sampling bias when planning online experiments.

4. Account for increased noise by increasing sample size

We suggest estimating a sample size based on previous data and to increase the estimated sample size by at least 33% to account for noise due to online testing. Not all sources of noise can be totally controlled and minimized by the experimenter. Therefore, the amount of data needed to be able to draw sound statistical inferences is likely higher compared to lab settings. Researchers might as well choose to increase the number of trials in their experiment. However, it is advisable to keep online studies short because long, and possibly boring, tasks may lead to an attrition of participant’s attention or increase the rate of participants who abort the study (Sauter et al., 2020).

5. Carefully check quality of data after the experiment

After the experiment, it is important to carefully check the recorded audio files, e.g., by listening to the files to check the audio quality and to control whether participants answered correctly. Additionally, researchers should inspect the files for differing file lengths within any one participant. Furthermore, we suggest checking the estimated voice onsets and to use an outlier screening procedure before analyzing the data. Of course the best way to check the quality of your data is to replicate your basic effect first.

### Open issues

#### Differing file lengths

We lost several data sets before data analysis due to different audio file lengths within single participants. In most cases the audio files stemming from the same participant had the same duration. However, there were cases where we encountered different file durations within the data stemming from the same participant. We thoroughly investigated this issue and did not find any correlation between differing file length and any of the software or hardware configurations we had logged (browser used, operating system used). We were not able to clarify whether recordings started late (which would be fatal to an accurate estimation of response time), or were cut in the end (which would be less problematic). We therefore took a conservative approach and replaced participants for whom differing file lengths occurred in more than 20% of the audio files. We hope that future research and software development will help to tackle this problem and thus make the online collection of precisely timed naming responses necessary for many language production paradigms more efficient.

#### Potentially poor audiovisual synchrony

Lack of audiovisual synchrony is a documented challenge for web-based experiments (see also Bridges et al., 2020; Reimers & Stewart, 2016). We cannot quantify to what degree the problem of poor audiovisual synchrony, a lag of differing duration between presentation of a picture and start of the audio file creation, existed in our experiment, too. The trial duration itself is predetermined via the specific parameters set while programming the experiment. It is logged in the respective logfiles and has a high reliability, i.e., the actual trial duration corresponds to the predefined trial duration. However, within each trial, several events need to be executed by the browser: a visual stimulus needs to be presented and an audio recording needs to be started. Therefore, audiovisual synchrony can be poor for two reasons. First, there may be delays between when the visual stimulus onset is requested and when it actually appears on screen and second, there may be delays between the recording request and when it actually starts. While there exist technical solutions to minimize the first problem, it is still a task for JavaScript developers to minimize the delays between the recording request and its start (Bridges et al., 2020). Without special technical equipment it is not possible to log how long it takes for a request to be executed and thereby to quantify the problem of audiovisual synchrony for each and every participant. One way to potentially quantify the problem of audiovisual synchrony would be to externally monitor participants’ screens for the appearance of a stimulus, displaying an audio signal like a beep immediately upon stimulus appearance from an external device which will then be recorded on the audio file. Later, a comparison of the latency difference between requested stimulus onset and audio file offset with the beep signal onset on the audio file in relation to the audio file offset needs to be done. Obviously, this is not possible when running experiments online. Therefore, a careful examination of the relative timing of events within a single trial is beyond the scope of this article while it may in principle be done (Gilbert & Minors, 2020). However, given our replication of a small effect of 20 ms we deem it reasonable that the problem of audiovisual synchrony can be neglected and reaction times can be estimated to a degree of accuracy that was sufficient for our purpose. For the future, we recommend monitoring the developments tackling the issue of audiovisual synchrony. For the time being, it should be kept in mind that even though options for voice recording are available and the software allows to define when and for how long an audio file is recorded, this does not necessarily guarantee that the timing is sufficiently well controlled for in order to draw inferences from voice onset latencies.

### Future avenues

One of the biggest advantages of conducting language production experiment online is that language production research will become less dependent on available lab space and thereby become more accessible. This will not only be helpful in midst of the COVID-19 pandemic with researcher having to move their experiments online. Furthermore, undergraduate students might be able to run their own small studies without using lab space. Participants from bigger and more diverse samples, even in remote areas, can be accessed more easily as long as they have Internet access. Hitherto understudied populations could be more readily featured in language production research – making the field less reliant on the classic WEIRD (= Western, educated, industrial, rich, Democratic) population (Henrich et al., 2010). Data from more diverse samples will be essential to test the validity of empirical findings in the language production literature.

With this study we provide a hands-on solution for running language production experiments online. With this proof of concept and alongside our suggestions that were derived from our experiences with implementing the experiments, we are confident that also other types of language production experiments, for example semantic blocking or the cumulative naming task, can be implemented online (Fairs & Strijkers, 2021; Stark, van Scherpenberg, Obrig, & Abdel Rahman, 2021). This will help to address many of the hitherto open questions in language production research (e.g., Abdel Rahman & Melinger, 2019; Bürki et al., 2020).

## Data Availability

The datasets generated during and/or analyzed during the current study are available in the Open Science Framework repository, https://osf.io/uh5vr/?view_only=229679aa33604aa2a5cb400eab620996.

## Footnotes

1 When asked whether German was their native language, one participant selected “No” while she had given the information of being a German native speaker on the recruiting platform Prolific. As careful screening of her audio files did not give any hint that German might not be her native language, we decided to keep this participant.

2 Researchers wishing to analyze data of future experiments with ANOVAs can find the results of F_1_/F_2_within-subjects ANOVAs including effect sizes in the online Supplementary Material B to use for a priori power analyses (https://osf.io/uh5vr/?view_only=229679aa33604aa2a5cb400eab62099).

## Declarations

### Code availability

The code for the experiments in this study as well as analysis scripts are available in the Open Science Framework repository, https://osf.io/uh5vr/?view_only=229679aa33604aa2a5cb400eab620996.
